# Branching coral morphology affects physiological performance in the absence of colony integration

**DOI:** 10.1098/rsbl.2022.0414

**Published:** 2022-12-07

**Authors:** Peter J. Edmunds, Kelly W. Johnson, Scott C. Burgess

**Affiliations:** ^1^ Department of Biology, California State University, 18111 Nordhoff Street, Northridge, CA 91330-8303, USA; ^2^ Department of Freshwater and Marine Ecology, Institute for Biodiversity and Ecosystem Dynamics, University of Amsterdam, Science Park 904, Amsterdam 1098 XH, The Netherlands; ^3^ Department of Biological Science, Florida State University, Tallahassee, FL 32306-4295, USA

**Keywords:** Scleractinia, ocean acidification, nubbins, fragments

## Abstract

For nearly 50 years, analyses of coral physiology have used small coral fragments (nubbins) to make inferences about larger colonies. However, scaling in corals shows that linear extrapolations from nubbins to whole colonies can be misleading, because polyps in nubbins are divorced of their morphologically complex and physiologically integrated corallum. We tested for the effects of integration among branches in determining size-dependent calcification of the coral *Pocillopora* spp. under elevated *P*_CO_2__. Area-normalized net calcification was compared between branches (nubbins), aggregates of nubbins (complex morphologies without integration) and whole colonies (physiologically integrated) at 400 versus approximately 1000 µatm *P*_CO_2__. Net calcification was unaffected by *P*_CO_2__, but differed among colony types. Single nubbins grew faster than whole colonies, but when aggregated, nubbins changed calcification to match whole colonies even though they lacked integration among branches. Corallum morphology causes the phenotype of branching corals to differ from the summation of their branches.

## 1. Introduction

Multiple animal taxa including bryozoans, ascidians, graptolites and anthozoans [1] exploit a colonial modular design to produce colonies from iterated modules [2,3]. A selective advantage of this design is the potential for indeterminate growth through the replication of modules of a constant size, each with the capacity to feed, respire and reproduce [4]. If modules remain physiologically independent, the metabolic rate of the colony should increase isometrically with the number of modules [2,5]. This principle has been exploited in experimental studies of colonial modular taxa because it underpins the tacit assumption that the biology of fragments is proportionately similar to that of whole colonies [6–8].

Most scleractinian corals exploit a colonial modular design, and the understanding of their biology has been built on 100 years of experiments with fragments [9], which were formalized as ‘nubbins’ in the 1970s [10]. In colonies that adhere to a colonial modular design and isometric scaling [2,5], physiological traits in nubbins should scale linearly to estimate trait values in larger colonies. With this rationale, experiments with nubbins have shaped how scleractinians are viewed as ecosystem engineers [11] in terms of calcification [6], photosynthesis [12] and nutrient recycling [13].

However, experiments with nubbins overlook evidence that big colonies are more than multiple nubbins [14], which is reflected in physiological traits that scale allometrically [15–17]. Larger colonies have lower mass-specific respiration and photosynthesis than smaller colonies [18], which is characteristic of unitary taxa [19]. Allometric scaling of calcification [17,18], the trait underpinning net community calcification on coral reefs [20,21], highlights the importance of understanding the extent to which the biology of nubbins reflects larger colonies. Nubbin physiology is likely to differ from that of larger colonies, because their polyps are physiologically disconnected from other parts of the colony, and they operate independent of ‘self-shading’ effects arising from interactions with structural elements inherent to colony design (e.g. branches) [22]. In branching corals, the physical and chemical conditions experienced by polyps in the interstices among branches are unlike those of ambient seawater [23,24], causing physiological processes in large colonies to differ from those of small colonies [25]. These effects are probably accentuated by confluent tissues and a common skeleton, which facilitate translocation of metabolites [26,27], the distribution of light [28], and the flow of mucus across the tissue [29]. Finally, polyp dimensions (e.g. expanded height) and biomass (i.e. tissue thickness) can adopt a variety of values in different colonies [15,30–32], which is a leading reason for colony phenotype to be an emergent property of corallum morphology and environmental conditions [25].

Despite the continued use of nubbins in coral biology, many studies with corals [15,16,17], and other colonial modular taxa [3], demonstrate the fallacy of isometric scaling in organisms exploiting this design. Branching pocilloporids are interesting to consider with respect to nubbin biology, as this taxon dominates Indo-Pacific reefs [33,34], and produces large colonies that are functionally unequal to small colonies [18,35]. Here we test the effects of colony size and integration among branches on calcification of *Pocillopora* spp. by manipulating the number of branches and integration among them to compare the response of nubbins, aggregates and colonies to seawater *P*_CO_2__. We focused on *P*_CO_2__ because it drives ocean acidification to which the response of corals, especially for traits like calcification, is being widely addressed using nubbins [8].

## 2. Methods

### (a) Overview

**Figure 1.**
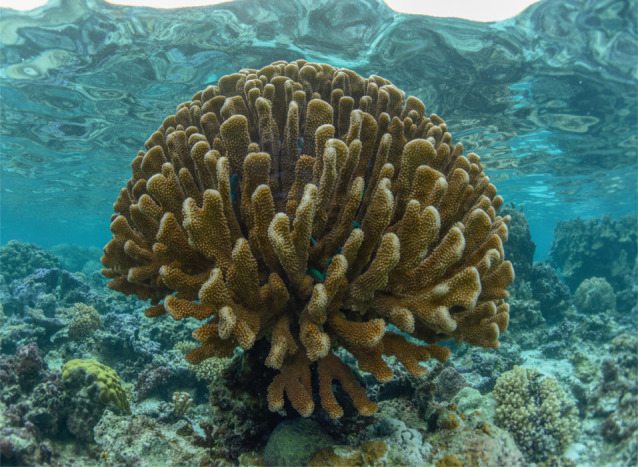
*Pocillopora* spp. at approximately 1.5 m depth showing branches and the tissue with which they are connected.

The experiment was completed with *Pocillopora* spp. (figure 1) from the fore reef of Moorea, French Polynesia, using trials completed from April to May of 2019 and 2022. Trials were conducted in 150 l tanks that were heated, mixed, chilled, illuminated and supplied with CO_2_ gas. We targeted *P. verrucosa* for collection based on morphology [36], although we probably worked with cryptic species [35,37] and, hereafter, we refer to our study organism as *Pocillopora* spp. Sampled corals were used to prepare single branches (nubbins), ‘artificial colonies’ (aggregates) of nubbins from the same genotype that are glued to a plastic base in an array creating the branch spacing of natural colonies (but lacking integration because the nubbins are not connected by tissue or skeleton) and intact colonies (colonies).

Following preparation, corals were placed in a 1000 l tank to adjust to laboratory conditions at approximately 29°C and a mean maximum photon flux density (PFD) of 511 ± 6 µmol photons m^−2^ s^−1^ (2019) or 563 ± 60 µmol photons m^−2^ s^−1^ (2022) (± s.e., *n* = 6). Corals remained in this tank for 3 (2019) or 9 days (2022) before being weighed and allocated to treatments. Treatment tanks were maintained at 28.9 ± < 0.1°C (ambient seawater in April 2019 was 30.0 ± 0.1°C [mean ± s.e., *n* = 30 days]) and illuminated at a mean maximum PFD of 561 ± 1 µmol photons m^−2^ s^−1^ (2019) or 484 ± 7 µmol photons m^−2^ s^−1^ (2022) (mean ± s.e., *n* = 200 and 48, respectively), on a 12 : 12 h light : dark photoperiod with ramping (electronic supplementary material).

### (b) Trial 1 (2019)

The experiment compared the effects of 414 µatm versus approximately 1041 µatm *P*_CO_2__ on corals at approximately 28.9°C. One nubbin and one paired aggregate of nubbins (sharing a host genotype), as well as one intact colony, were randomly allocated to each of the four tanks at ambient or elevated *P*_CO_2__. The elevated *P*_CO_2__ represented a pessimistic projection for the end of the century (RCP 8.5 [38,39]).

On 5 April, corals (*n* = 16 genotypes, approximately 12 cm diameter) were collected from 10 m depth, prepared as nubbins, aggregates and colonies, and transferred to the 1000 l tank to adjust to laboratory conditions. On 8 April, they were buoyant weighed (electronic supplementary material) and randomly placed in the tanks on 9 April. Tanks were illuminated at a mean maximum PFD of 561 ± 1 µmol photons m^−2^ s^−1^ (± s.e., *n* = 200) on a 12 : 12 h light : dark photoperiod with ramping. The corals were moved daily within each tank, and after 21 days, were buoyant weighed, and their areas determined by wax dipping [40]. Changes in buoyant weight were converted to mass representing net calcification and standardized to area and time (mg cm^−2^ d^−1^).

### (c) Trial 2 (2022)

In 2022, the experiment was repeated, but the design slightly changed due to logistical constraints. On 5 April, corals (*n* = 24 genotypes, approximately 12 cm diameter) were collected from 10 m depth when ambient seawater temperature was approximately 29.0°C. Corals were prepared as nubbins, aggregates and colonies.

Prepared corals were transferred to the approximately 1000 l tank on 6 April, and on 13 April they were buoyant weighed and randomly allocated to three tanks at each of ambient *P*_CO_2__ and elevated *P*_CO_2__ at approximately 29.0°C. Two nubbins, two aggregates and two intact colonies were placed in each tank. Tanks were illuminated at a mean maximum PFD of 484 ± 7 µmol photons m^−2^ s^−1^ (±s.e., *n* = 48) on a 12 : 12 h light : dark photoperiod with ramping. The corals were moved daily within each tank, and after 21 days, were buoyant weighed, and their areas were determined by wax dipping [40]. Changes in buoyant weight were converted to mass representing net calcification and standardized to tissue area and time (mg cm^−2^ d^−1^).

### (d) Analyses

The effects of colony type (nubbins, aggregates or colonies) and *P*_CO_2__ on net calcification were modelled using a Gaussian linear mixed effects model implemented in *R* using the *glmmTMB* package [41]. Year and tank were treated as a single random effect, and the interactive and additive fixed effects of colony type and *P*_CO_2__ were assessed using log-likelihood ratio tests with α = 0.05. Pairwise differences among colony types were calculated using the estimated marginal means using the *emmeans* package in R. *p*-Values were adjusted for multiple comparisons using the Tukey method.

## 3. Results

**Figure 2.**
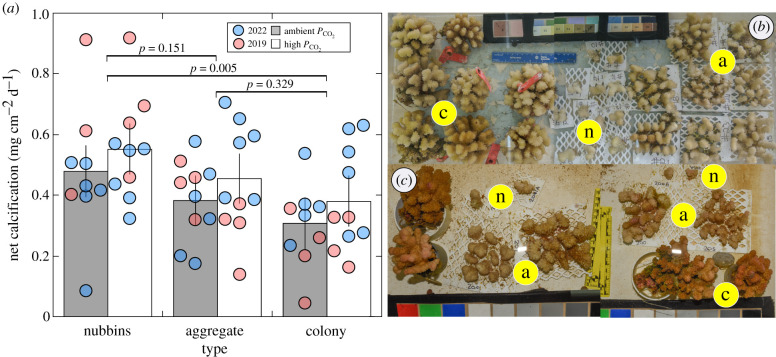
Net calcification of *Pocillopora* spp. (*a*) at approximately 400 µatm or approximately 1000 µatm *P*_CO_2__ in 2019 (red) and 2022 (blue). Bars show the mean and vertical lines show the 95% confidence interval (*n =* 9–10 for all treatments). Net calcification differed between nubbins and colonies (horizontal lines and *p*-values). Photographs show corals in 2019 (*b*) and 2022 (*c*), with lowercase letters marking nubbins (n), aggregates (a) and colonies (c).

The conditions in the tanks are described in the electronic supplementary material. In 2019, the corals maintained positive net calcification ranging from 0.05 mg cm^−2^ d^−1^ to 0.92 mg cm^−2^ d^−1^ (figure 2*a*). In 2022, one coral lost weight through breakage and was excluded from the analysis, and the remaining corals calcified at between 0.08 mg cm^−2^ d^−1^ and 0.71 mg cm^−2^ d^−1^ (figure 2*a*).

Across two trials, net calcification differed among colony types (χ^2^ = 9.980, d.f. = 2, *p* = 0.007), but not between *P*_CO_2__ treatments (χ^2^ = 2.297, d.f. = 1, *p* = 0.130), and there was no interaction between the main effects (χ^2^ = 0.075, d.f. = 2, *p* = 0.963). Net calcification in single nubbins was 0.171 mg cm^−2^ d^−1^ (0.069 to 0.274, 95% CI) higher than that for intact colonies (*t* = 3.290, d.f. = 52, *p* = 0.005) (figure 2). However, aggregates of nubbins had the same net calcification as intact colonies (*t* = 1.439, d.f. = 52, *p* = 0.329). Furthermore, net calcification was 0.097 (−0.004 to 0.198, 95% CI) mg cm^−2^ d^−1^ lower for aggregates of nubbins compared to single nubbins, but this effect could not be statistically distinguished from zero (*t* = 1.892, d.f. = 52, *p* = 0.151).

One nubbin (in 2022) and one colony (in 2019) calcified at less than or equal to 0.09 mg cm^−2^ d^−1^, which identified them as biological outliers. When these values were excluded, net calcification in aggregates of nubbins was significantly lower than in single nubbins (by 0.121 (0.025 to 0.216, 95% CI) mg cm^−2^ d^−1^) (*t* = 2.480, d.f. = 50, *p* = 0.043). All other results remained qualitatively similar (electronic supplementary material, figure S1).

## 4. Discussion

A colonial modular design is central to the success of scleractinians, because it allows indeterminate size [42], and the morphologically complex colonies that define this taxon. Through asexual proliferation, polyps remain connected through their gastrovascular cavities [43,44], a common skeleton and surface mucous [29], allowing networks of connected polyps to achieve properties that are more than the sum of the parts. The early notion that this design facilitates isometry [42,45] has been replaced by an expectation of allometry that is consistent with the contemporary understanding of scaling in this taxon [17,18], as well as the mechanisms integrating polyps [28,29] and determining their size [15,46]. Nonetheless, the functional implications of corallum morphology remain incompletely known. Here we show that closely spaced branches of *Pocillopora* spp., without tissue or skeletal connections among them, display the physiological phenotype of intact colonies, with trait values higher when separated. This outcome highlights the importance of bio-physical coupling between corals and the environment (versus the intrinsic consequences of coloniality) in determining phenotype, although currently we cannot reject an alternative hypothesis, that the growth of isolated nubbins is elevated through repair of their fractured base and calcification stimulated by the lack of shading by adjacent branches [21]. Our results have important implications for the use of nubbins to make inferences about the performance of whole corals and coral reefs in an anthropogenically disturbed world.

Scientific understanding of scleractinian biology has long reflected a duality of approaches, one addressing intrinsic aspects of their design, including coloniality, symbiosis, and the cnidarian bauplan [1], and the other, the ways through which this design interacts with the environment [15,47]. While the functional consequences of coral skeletons have remained enigmatic, recent research has focused on intrinsic features to understand the properties of coralla. Since, for example, light is channelled through coral skeletons and tissue [28], metabolites and symbionts are moved within the gastrovascular cavity [44], and mucous streams among polyps [29], it is reasonable to expect that skeletal and tissue continuity among branches contributes to the colony phenotype. For *Pocillopora* spp., these effects appeared subordinate to the consequences of closely spacing branches, causing aggregates of nubbins to behave as intact colonies (figure 2*a*), particularly when two outlying corals that barely grew were excluded (electronic supplementary material, figure S1). While the lack of effect of *P*_CO_2__ on calcification is inconsistent with previous studies [8], this outcome is likely to reflect the limitations of short experiments in detecting small treatment effects (e.g. fig. 2 in [8]).

In Moorea, spawning *Pocillopora* spp. represent an assemblage of cryptic species with differential bleaching susceptibility [35]. In *Pocillopora* spp., we have shown that morphological taxonomy [36] is a poor indicator of species identity [35,37], and therefore, it is possible that both of our trials contained more than one species. Since at least some of the species in this complex are physiologically unequal as demonstrated by contrasting bleaching susceptibility [35], it is also possible that they differentially translate colony integration into emergent aspects of colony performance. While this physiological landscape remains to be explored, the outcome is unlikely to affect the present conclusions that rely more on bio-physical coupling between the environment and corallum structure, than intrinsic biology *per se*. Any differences among species would create variance in our result, rather than bias any treatment affects, since all colonies were randomly allocated to treatments.

The outcome of our experiment is ecologically significant because net calcification underpins the ecosystem engineering role of scleractinians [48] and generates the coralla that are the quintessence of coral reefs. After decades of research on coral calcification [49], and recent research underscoring the elegance of coloniality [17,28], integration with colonies [29] and the expanding realm of symbiosis and the microbiome in this taxon [50], it is reasonable to expect these features might have roles in creating the physiological phenotypes of branching corals. While such effects undoubtedly are occurring, the present study shows that simply placing nubbins close together creates a phenotype common to a natural colony. Rather than integrative effects *per se*, this outcome underscores the interactions of branching coralla with flowing seawater [15,51], and the creation of physical (e.g. light and flow speed) and chemical (e.g. nutrients, organic carbon, etc.) microenvironments between branches [24,52] in determining how corals (figure 1) function in the complex physical world of a coral reef.

## Data Availability

The data are provided in the electronic supplementary material [53].
